# Diagnostic value of urinary Tamm-Horsfall protein and 24 h urine osmolality for recurrent calcium oxalate stones of the upper urinary tract: Cross-sectional study

**DOI:** 10.1515/med-2023-0848

**Published:** 2023-11-14

**Authors:** Daocheng Fang, Yuanyuan Hu, Chao Wang, Chunhua Tang

**Affiliations:** Department of Urology, Shanghai Songjiang District Central Hospital, Shanghai, 201600, China

**Keywords:** calcium oxalate, Tamm-Horsfall protein, urine osmolality, recurrence

## Abstract

We aimed to investigate the diagnostic value of urinary Tamm-Horsfall protein (THP) and 24 h urine osmolality for recurrent calcium oxalate (CaOx) stones. Clinical data of 120 patients with upper urinary tract stones admitted to our hospital between January 2020 and January 2022 were retrospectively reviewed. Patients were divided into recurrence (53 patients) and non-recurrence (67 patients) groups based on postoperative stone recurrence. Meanwhile, 50 healthy patients were selected as the control group. Urinary THP levels, 24 h urine osmolality, and biochemical indices were compared between the three groups; their diagnostic values for stone recurrence were evaluated using receiver operating characteristic (ROC) curves. Urinary THP, 24 h urine osmolality, and biochemical indices were significantly higher in the recurrence group than in the non-recurrence and control groups (*P* < 0.05). The 24 h urine osmolality was positively correlated with urinary oxalic acid and calcium excretion. ROC curve analysis showed that optimal cutoff values of urinary THP and 24 h urine osmolality for the diagnosis of stone development were ≥27.01 mg/L and ≥577.69 mOsm/(kg H_2_O), respectively. Furthermore, these indices combined significantly improved the accuracy of diagnosis. Urinary THP and 24 h urine osmolality were higher in patients with recurrent CaOx stones. Detection of both parameters combined can accurately diagnose stone recurrence.

## 1 Introduction

Urolithiasis is a common disease in urology that has shown an increasing incidence and recurrence over recent years [1,2]. The most common stone composition is based on calcium salts (mostly calcium oxalate [CaOx]); this form of stone accounts for the highest proportion of all stones [3]. Frequent recurrence of urinary calculi can cause many problems, such as serious complications and high treatment costs [4]. Urolithiasis is a disease formed under the combination of many factors such as multi-gene, environment, diet, race, and abnormal metabolism. Therefore, the selection of sensitive indicators to diagnose the recurrence of CaOx stones is of great significance in preventing urinary calculi in patients. At present, there are many studies on urinary calculi indexes, but these indexes are not specific, high cost, and cannot be used in clinical practice. It is a feasible direction to detect stone-related metabolites in the human body for the early warning of urinary calculi, and Tamm-Horsfall protein (THP), as a specific product of the urinary system, has gradually attracted significant attention as this protein exhibits a certain relationship with the formation of CaOx stones [5].

The metabolic assessments of patients with urinary calculi include analysis of stone and 24 h urine components, serum chemical examination, and so on, in which low urine volume and high urine concentration are important factors for the occurrence of urinary stones [6]. The urinary concentration of stone formation is affected by the daily urine volume; increasing the daily fluid intake is an important factor in preventing stone recurrence [7]. Furthermore, 24 h urine osmolality is an appropriate biomarker for determining the optimal fluid intake for an individual. Urinary THP and 24 h urine osmolality are easy to collect and non-invasive and are, therefore, potential clinical diagnostic indicators. However, very few studies have investigated the ability of these parameters to diagnose the recurrence of CaOx stones. Therefore, in this study, we investigated the diagnostic value of urinary THP and 24 h urine osmolality for recurrence in patients with CaOx stones.

## 2 Methods

### 2.1 Study population

We selected a total of 120 patients with urinary calculi who were treated in our hospital between January 2020 and January 2022. The inclusion criteria were as follows: (a) patients diagnosed with upper urinary tract urolithiasis based on history, signs, and examination findings; (b) age ≥18 years; (c) patients with unilateral urinary stones; (d) postoperative stone composition analysis confirmed the presence of CaOx stones; and (e) postoperation abdominal computerized tomography (CT) showed no residual stones. The exclusion criteria were as follows: (a) urinary infection, (b) urinary malformation, (c) serum creatine greater than 133 umol/L, (d) diabetes, (e) hypertension, (f) hyperlipidemia, (g) obesity (BMI ≥28 kg/m^2^), and (h) taking diuretics. Fifty healthy patients during the same period were selected as the control group; the inclusion criteria for the control group were as follows: (a) no history of urinary calculi and no family history and (b) age ≥18 years. The exclusion criteria were the same as those of the stone group. 


**Ethical approval and consent to participate:** Informed consent of patients was obtained for this study.
**Human ethics:** This study has been filed by the Medical Ethics Committee of Shanghai Songjiang Central Hospital.

### 2.2 Methodology

All patients followed a recommended diet that limited oxalate-rich foods, reduced animal protein and salt, and increased the intake of fruits, vegetables, and fluids (≥2 L/day). A 6-month postoperative follow-up was performed for patients in the stone group, and their urinary tract ultrasound or abdominal CT and urine routine were regularly reviewed. According to postoperative follow-up, the patients were divided into a recurrence group and a non-recurrence group; 50 healthy patients during the same period were selected as the control group. In the recurrence group, a urine test was conducted immediately after the recurrence of stones, while in the non-recurrence group, the time of the urine test was 6 months after the operation. The urinary THP, 24 h urine osmolality, and biochemical indices were tested.

The method for detecting THP in the urine was as follows. First, 5 mL of midstream urine was collected from each patient in the three groups in the morning. Samples were centrifuged at 3,000 r/min for 20 min and stored in a refrigerator for subsequent analysis. Enzyme-linked immunosorbent assays (ELISA) were used for detection. ELISA kit was purchased from Shanghai Qiaoyu Biological Co., Ltd (China). The specific detection operation was performed in strict accordance with the manufacturer’s instructions. The wavelength was adjusted to 450 nm to detect the absorbance of each well, the mean value was taken after three measurements, a standard curve was plotted according to the concentration of each standard and the absorbance, and then the concentration of each sample to be tested was calculated.

The detection methods for 24 h urine osmolality and biochemical indices were as follows. Urine was collected from each patient over a 24 h period (07:00 on the morning of Day 1 to 07:00 on the morning of Day 2); the first excreted urine was discarded. Boric acid was added in advance for antisepsis treatment (1% boric acid). The volume of urine was accurately measured and recorded and sent to the laboratory department of our hospital for the analysis of 24 h urine osmolality, oxalic acid, and calcium.

### 2.3 Statistical analysis

Normally and non-normally distributed continuous variables are expressed as mean ± standard deviation and median (interquartile spacing), respectively. One-way analysis of variance was used for comparisons among multiple groups, and *t*-tests were used for comparisons between groups. Counting variables are expressed as numbers and percentages, and the Chi-squared test was used for comparisons. The relationship between 24 h urine osmolality and urinary THP was assessed by Pearson correlation coefficients. Receiver operating characteristic (ROC) curves were constructed to identify the UOsm cutoff value that would confer optimal sensitivity and specificity for the diagnosis of recurrence. All statistical analyses were performed using SPSS 22.0 software. *P* < 0.05 was statistically significant.

## 3 Results

### 3.1 Comparison of general data among the three groups

There was no significant difference in the general data when compared between the three groups (*P* > 0.05), as shown in Table 1, indicating that the basic conditions of the three groups of patients in this study were comparable.

**Table 1:** Comparison of basic information in the recurrence, non-recurrence, and control groups (mean ± SD)

Indicators	Recurrence group	Non-recurrence group	Control group	*F*/*X* ^2^	*P* value
Number of cases	53	67	50	0.375	0.708
Age (years)	46.54 ± 4.50	41.37 ± 7.65	39.19 ± 8.12	0.947	0.331
**Gender (cases [%])**					
Male	32 (60.4)	39 (58.2)	25 (50.0)	0.210	0.646
Female	21 (39.6)	28 (41.8)	25 (50.0)	0.070	0.792
**Location of recurrence (cases [%])**					
Kidney	40	/	/	/	/
Ureter	13	/	/	/	/

### 3.2 Comparison of 24 h urine biochemical indices among three groups

The 24 h urine biochemical indices of patients in the recurrence group were significantly higher than those in the non-recurrence group and the control group (*P* < 0.05). There was no significant difference between the non-recurrence group and the control group (*P* > 0.05), as shown in Table 2.

**Table 2:** 24 h urinary biochemical indices of patients in each group (mean ± SD)

Group	24 h urinary calcium (mg/24 h)	24 h urinary oxalic acid (mg/24 h)
Recurrence group	158.42 ± 62.13	48.41 ± 11.22
Non-recurrence group	126.27 ± 51.22	32.36 ± 7.38
Control group	123.12 ± 53.45	32.34 ± 5.26
*F*	13.013	6.121
*P* value	0.021	0.032

### 3.3 Comparison of urinary THP and 24 h urine osmolality between the three groups

The levels of urinary THP and 24 h urine osmolality in the recurrence group were significantly higher than those in the non-recurrence group and the control group (*P* < 0.05). There was no significant difference in the levels of urinary THP and 24 h urine osmolality when compared between the non-recurrence group and the control group (*P* > 0.05), as shown in Table 3.

**Table 3:** 24 h urinary osmolality and urinary THP levels of patients in each group (mean ± SD)

Group	24 h urinary osmolality [mOsm/(kg H_2_O)]	Urinary THP (mg/L)
Recurrence group	590.49 ± 25.56	28.36 ± 1.97
Non-recurrence group	564.73 ± 12.33	26.23 ± 1.17
Control group	560.65 ± 18.29	26.11 ± 1.84
*F*	11.452	5.748
*P* value	0.026	0.039

### 3.4 Correlation between urinary THP and 24 h urine osmolality

**Figure 1:**
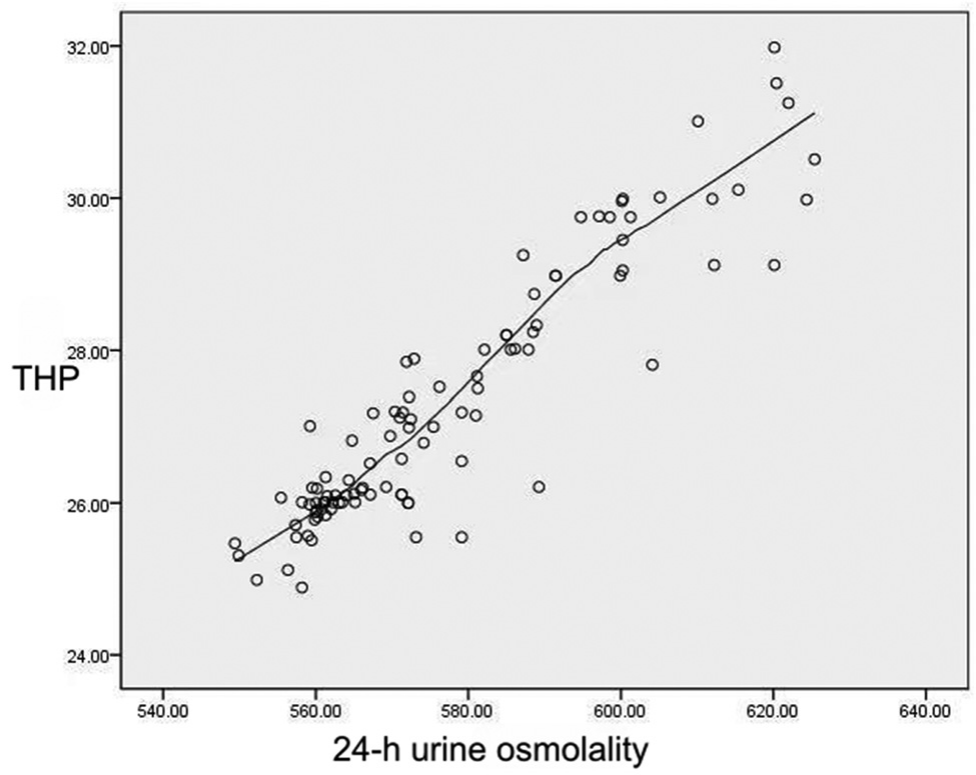
Scatter distribution of urinary THP and 24 h urine osmolality.

Analysis showed that there was a significant correlation between urinary THP and 24 h urine osmolality (*r* = 0.662, *P* < 0.05). Scatter diagram analysis showed that there was a certain linear relationship between these parameters, as shown in Figure 1.

### 3.5 ROC curve analysis of urinary THP, 24 h urine osmolality, and combined testing for the diagnosis of stone recurrence

**Figure 2:**
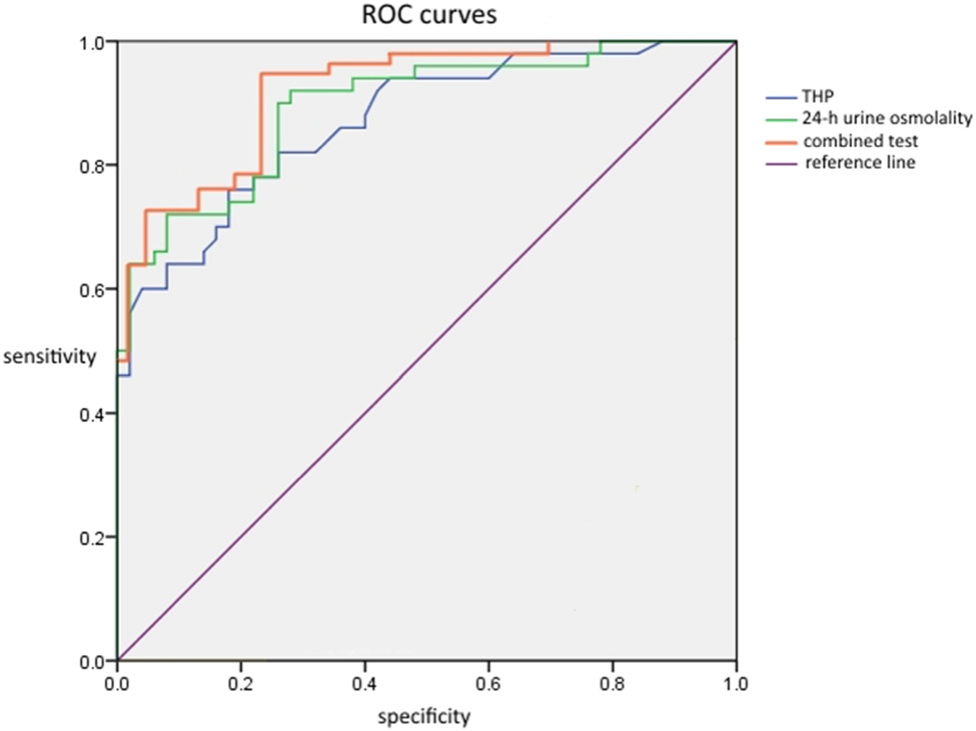
ROC curves for urinary THP, 24 h urine osmolality, and combined detection.

Urine THP and 24 h urine osmolality were analyzed by binary logistic regression to determine prediction probability and joint detection. ROC curves were created for all three methods; the area under the curve (AUC) was 0.850, 0.871, and 0.912, respectively. Combined detection yielded the best diagnostic performance. According to the Youden index, the optimal cutoff points for urinary THP and 24 h urine osmolality for the diagnosis of stone recurrence were 27.01 mg/L and 577.69 mOsm/(kg H_2_O), as shown in Table 4 and Figure 2.

**Table 4:** Efficacy of urinary THP, 24 h urine osmolality, and combined detection

Indicators	AUC	Cutoff value	Sensitivity (%)	Specificity (%)
Urinary THP	0.850	27.01	76.56	82.47
24 h urinary osmolality	0.871	577.69	82.31	90.10
Joint testing	0.912	0.92	92.01	86.55

## 4 Discussion

Urolithiasis is one of the most common diseases in urology. The specific pathogenesis of this condition is ominous and is thought to be caused by genetic and environmental factors. The composition of urinary stones is complex, although most are CaOx stones [3]. Due to the high incidence of this condition, patients can be hospitalized multiple times due to stone recurrence, thus exerting pressure on both the patients and the medical and health systems. For patients with urinary stones, the prevention of stone recurrence is as important as the initial treatment. Therefore, we need to develop a non-invasive, convenient, and sensitive early marker to diagnose the risk of urolithiasis recurrence in patients, and carry out subsequent dietary and lifestyle interventions through risk prediction. The development of such methods would provide a valuable contribution to the field of urolithiasis.

The formation of urinary stones *in vivo* is affected by various mechanisms, and many proteins are known to be involved in the inhibition or promotion of stone formation, such as osteopontin and THP. THP is a matrix protein of urinary tubular formation and plays a crucial role in the formation of urinary calculi because THP aggregates are also one of the matrix organic components of urinary calculi [5]. In healthy individuals, a small amount of THP will be discharged into the urine. When kidney injury is caused by various reasons such as obstruction, inflammation, or autoimmune diseases, the content of THP in the urine will increase, and the greater the degree of kidney injury, the higher the concentration of THP in the urine. However, some studies have suggested otherwise [8,9]. Therefore, the relationship between renal function injury and THP remains to be further studied. The same THP has a dual effect on the growth and aggregation of CaOx crystals, which is affected by many factors [5]. The interference effect of urinary THP on the adhesion of other ions on the surface of CaOx crystal has been studied previously, and a reduction in the maximum negative potential on the crystal surface has been found to promote crystal aggregation. When the concentration of urinary THP increases, the effect of THP on the promotion of crystal aggregation is more obvious, which is strengthened as the ionic strength increases [9,10]. Related studies showed that crystallization in the kidney also increased and that the expression levels of THP increased significantly [11].

For most patients, the etiology of urolithiasis may be due to excessive urinary excretion of CaOx and urate or to the inadequate excretion of citrate, magnesium, and potassium, among others. Of these factors, inadequate urine dilution appears to be the most important and one of the most common. Low urinary volume and high urinary concentration are also considered to be important factors for urinary calculi [6]. Since the urine concentration for stone formation is strongly influenced by the daily urine volume, increasing the daily fluid intake is an important factor in preventing stone recurrence [7]. Although current guidelines recommend the intake of at least 2 L of fluid per day, other factors (such as hidden water loss and water contained in food and the composition of the fluid ingested) can also affect urinary volume [12]. Urinary volume and concentration reflect the body’s hydration status, which maintains water balance at different water intake and loss levels. Therefore, an indicator representing the hydration status of patients is needed to diagnose stone recurrence and thus guide the optimal diet of patients. The guidelines for urolithiasis developed by the European Association of Urology recommend urinary specific gravity as an indicator; this should be maintained below 1.010 [13]. Generally speaking, urine-specific gravity has a certain correlation with urine osmolality. However, urine-specific gravity is susceptible to interference from many factors, such as pH and solute composition. In contrast, urine osmolality is independent of the molecular weight of the dissolved material, the volume and density of the particles in the urine, and only the number of solute particles in the urine, thus reflecting the concentration of the solute in the urine. A study of the relationship between urine-specific gravity and the *in vitro* permeability of urine samples with different compositions suggested that urine-specific gravity may be higher or lower than urine osmolality under different clinical conditions [14]. Therefore, 24 h urine osmolality is an appropriate biomarker with which to measure an individual’s appropriate fluid intake [15]. In a previous study, 24 h urine samples were evaporated rapidly under strict conditions. The urine samples were then evaporated to the metastable limit of insoluble salts, thus resulting in spontaneous crystallization. It was found that the urine in the stone group evaporated by 44.2% and that the urine in the non-stone group evaporated by 72.1%. Crystal aggregation occurred, thus suggesting that the urine osmotic pressure in the stone group was higher and the urine was easier to crystallize [16].

The results of this present study showed that both urinary THP and 24 h urine osmolality in the recurrence group were higher than those in the non-recurrence group and the control group. We also found that there was a certain correlation between urinary THP and 24 h urine osmolality; our findings were similar to those published previously [17,18]. As a protein secreted by the ascending branch of the renal tubular medullary loop and the distal tubular curvature, THP plays an important role in regulating the function of the thick segment of the ascending branch of the normal renal medullary loop [5]. Increased levels of THP will lead to an increase in the solute concentration in urine, which may lead to an increase in urinary osmolality. We found that within a certain range when the level of THP increased, the 24 h urinary osmolality also increased; the difference between the recurrence and non-recurrence groups indicated that THP was associated with the recurrence of urinary stones. The results of ROC curve analysis showed that the combination of urinary THP and 24 h urinary osmolality had a higher diagnostic efficiency and specificity for the recurrence of CaOx stones than those of urinary THP or 24 h urinary osmolality alone. Urinary osmolality is easily affected by many factors, such as diet and metabolism [14], while THP in human urine can be affected by other diseases of the urinary system. Therefore, our data suggested that the combined detection of these two parameters can accurately diagnose the recurrence of CaOx stones.

However, there are some limitations to our study that need to be considered. First, this was a retrospective study carried out in a single center. Second, although patients were told what to eat, there was no guarantee that they all followed our instructions. Furthermore, because urinary stones are affected by many factors, it is possible that our data cannot be generalized to other populations. Despite these limitations, to the best of our knowledge, this is the first study to evaluate the value of urinary THP with 24 h urinary osmolality for diagnosing recurrence in patients with CaOx stones, thus providing new evidence for the diagnosis of recurrence. Compared with other common indicators of the recurrence of kidney stones, the two indicators in this study have the advantages of being non-invasive and easy to measure, having relatively high accuracy, as well as not being easily interfered with.

## 5 Conclusion

This study is the first to investigate the diagnostic value of urinary THP combined with 24 h urine osmolality for the recurrence of CaOx stones. We believe that because these two indicators are easy to collect, they have a certain potential for clinical application.
